# Knowledge Exchange and Discovery in the Age of Social Media: The Journey From Inception to Establishment of a Parent-Led Web-Based Research Advisory Community for Childhood Disability

**DOI:** 10.2196/jmir.5994

**Published:** 2016-11-11

**Authors:** Dianne J Russell, Jennifer Sprung, Dayle McCauley, Olaf Kraus de Camargo, Francine Buchanan, Roman Gulko, Rachel Martens, Jan Willem Gorter

**Affiliations:** ^1^ CanChild Center for Childhood Disability Research McMaster University Hamilton, ON Canada; ^2^ School of Rehabilitation Science McMaster University Hamilton, ON Canada; ^3^ Department of Pediatrics McMaster University Hamilton, ON Canada; ^4^ Institute of Health Policy, Management and Evaluation University of Toronto Toronto, ON Canada

**Keywords:** knowledge exchange, research engagement, collaborative research, scientific collaboration, Web-based community, social media, Facebook, childhood disability, patient and public involvement(PPI)

## Abstract

**Background:**

Efforts to involve parents and families in all aspects of research, from initiating the question through to dissemination and knowledge exchange, are increasing. While social media as a method for health communication has shown numerous benefits, including increasing accessibility, interactions with others, and access to health care information, little work has been published on the use of social media to enhance research partnerships.

**Objective:**

Our objective was to describe the development and evaluation of a Web-based research advisory community, hosted on Facebook and connecting a diverse group of parents of special needs children with researchers at *CanChild Centre for Childhood Disability Research*. The goal of this community is to work together and exchange knowledge in order to improve research and the lives of children and their families.

**Methods:**

The Web-based Parents Participating in Research (PPR) advisory community was a secret Facebook group launched in June 2014 and run by 2 parent moderators who worked in consultation with *CanChild*. We evaluated its success using Facebook statistics of engagement and activity (eg, number of posts, number of comments) between June 2014 and April 2015, and a Web-based survey of members.

**Results:**

The PPR community had 96 participants (2 parent moderators, 13 researchers, and 81 family members) as of April 1, 2015. Over 9 months, 432 original posts were made: 155 (35.9%) by moderators, 197 (45.6%) by parents, and 80 (18.5%) by researchers. Posts had a median of 3 likes (range 0-24) and 4 comments (range 0-113). Members, rather than moderators, generated 64% (277/432) of posts. The survey had a 51% response rate (49/96 members), with 40 (82%) being parent members and 9 (18%) being researchers. The initial purpose of the group was to be an advisory to *CanChild*, and 76% (28/37) of parents and all the researchers (9/9) identified having an impact on childhood disability research as their reason for participating. A total of 58% (23/40) of parents and 56% (5/9) of researchers indicated they felt safe to share sensitive or personal information. While researchers shared evidence-based resources and consulted with families to get guidance on specific issues, there was an unexpected benefit of gaining an understanding of what issues were important to families in their daily lives. Parents felt a sense of belonging to this community where they could share their stories but also wanted more researcher participation and clarity on the purpose of the group.

**Conclusions:**

The PPR community grew from inception to an established community with active engagement and knowledge exchange. Both parents and researchers described valuable experiences. Researchers should consider social media as a means of engaging families in all phases of research to ensure that research and its outcomes are meaningful to those who need it most.

## Introduction

Families with children with disabilities and medical complexity constitute approximately 4.6% of Canada’s pediatric population under the age of 15 years [1]. The growth in this population over the past 20 years has driven an increase in childhood disability research. Historically, in childhood disability research, applied health researchers seeking to directly influence clinical practice have worked collaboratively with individuals responsible for making relevant clinical, health, and social policy decisions and allocating resources [2]. However, over the past 5 to 10 years, efforts to actively involve families and patients in research have been increasing. Rosenbaum, in a position piece on family-centered research, identified “how much richer our studies have become with the active input of families and parents and thoughtful critics during the development of projects” [3]. Involving families in research is believed to improve service delivery, patient experience, and patient outcomes [4]. Input from families generates research questions that are targeted at family needs, which are not always aligned with the priorities of researchers. Efforts to identify high-priority questions in cerebral palsy research found that, although there was considerable overlap between what clinicians and families considered key research topics, some topics that families identified as important were not considered important by clinicians. The researchers discovered that social issues and effective alternative therapies were not of interest to clinicians but were important to families as they related to daily function and activity [5].

In addition to the growing amount of support for the inclusion of families in the research process [2,3,6], the expectations of funding agencies that patients and families be included are also increasing [7-9]. Although the importance of and need for engagement have been acknowledged, little evidence exists about the best way to actively engage families to provide input that is valuable to clinicians and researchers [6,10,11]. Research conducted into engaging families in research has highlighted several barriers that limit the ability of families to participate in research and be fully engaged. From a researcher’s perspective, these barriers may include a desire to maintain control, unwillingness to consider parents as equals in terms of contributions and competence, and time and cost limitations. From a consumer’s perspective, these barriers may include time, difficulty accepting and transitioning into a new role, and lacking knowledge or the confidence to contribute [4].

Social media have received increased attention over the past 10 years as a means of connecting and improving health communication. Social media platforms such as Facebook and Twitter are free, and provide quick and accessible methods to access information and engage with other stakeholder groups. While 52% of online adults use multiple social media sites in the United States, 71% use Facebook, which remains the most popular site for those who use only one and overlaps significantly with other platforms [12]. In a systematic review, Moorhead et al identified the benefits of social media (including Wikipedia, YouTube, Facebook, and virtual game and social worlds) for health communication as (1) increased interactions with others, (2) more available, shared, and tailored information, (3) increased accessibility and widening access to health information, (4) peer, social, and emotional support, (5) public health surveillance, and 6) the potential to influence health policy [13]. Limitations were mainly related to concerns about reliability of information, confidentiality, and privacy. Of the 98 research studies included in the review by Moorhead et al, 13 were using Facebook as a means of increasing awareness and communicating about a range of topics (eg, concussion, diabetes, breast cancer, attention-deficit/hyperactivity disorder) [13]. Facebook has also been used as part of a social media campaign intended to raise awareness for Hirschsprung disease and to connect and engage families affected by this rare condition [14]. While reach and responsiveness are considered strengths of social media usage, other studies have reported benefits of creating smaller communities. In particular, a primary care maternity clinic in Finland provided its clients with a Web service containing social media tools similar to those of Facebook, in order to foster a support network for its members [15]. The participating mothers reported that one factor that increased their feelings of belongingness was the fact that membership was strictly limited to clients of the same maternity clinic. This closed network positively affected the mothers’ levels of trust and increased their willingness to discuss intimate issues.

While describing management strategies for online health communities, Young proposed a community life cycle that consists of 4 stages: inception, establishment, maturity, and mitosis [16]. Each stage is characterized by various milestones, and monitoring a community’s growth can facilitate progression through these stages. The inception stage is the first stage that starts as soon as an organization begins to engage potential members. The primary focus during this stage is to make connections and build a core group of active members. Engagement at this time is limited, with only 0% to 50% of activity initiated by community members. The establishment stage comes next and begins when community members generate more than 50% of the activity and ends when they generate most (90%) of the growth and activity. The primary focus of this stage is establishing a sense of community by acknowledging the contributions of members and encouraging further participation and engagement. The maturity stage begins when more than 90% of community activity and growth is generated by its members. During this stage, the size of the community reaches its critical mass and the sense of community is well established. Although communities at this stage are considered self-sustaining, management is still needed. The final stage, known as the mitosis stage, begins when the community becomes largely self-sustaining and ends when activity and growth begin to negatively affect the sense of community. This is a critical stage, as successful communities run the risk of becoming too large and active, subsequently triggering member disengagement. Community monitoring is essential at this stage, as managers may witness the emergence of special interest groups and community subsets. These subgroups have the potential to split off to create splinter groups and begin the community life cycle once more.

We describe the development and evaluation of a Web-based research advisory committee hosted on Facebook and connecting a diverse group of parents of special needs children with researchers at *CanChild* at McMaster University in Hamilton, Ontario, Canada. The goal of establishing this parent-researcher community was to work together and exchange knowledge in order to improve research and the lives of children with special needs and their families. We describe the first year of our online community, during which we have moved from inception to an established community.

## Methods

### Building the Community

Based on *CanChild* ’s knowledge translation strategic plan [17], *CanChild* planned on developing a research advisory group to facilitate active engagement from family members. The purpose of the group would be to exchange knowledge on project planning, research direction, the current state of special needs parenting, supports, and services, as well as how to translate research knowledge to best serve parents and youth living with disability. The original vision for our research advisory group was to bring together youth and young adults with disabilities, family members, and researchers for quarterly meetings (either in person or via teleconference) to facilitate the research direction. In early discussions (October 2012) related to the development of this group *,* a parent (JS) proposed the idea of a parent advisory community hosted on Facebook (Facebook, Inc, Menlo Park, CA, USA). It was thought that a virtual group would allow greater involvement from families and researchers (both geographically and categorically) and more instantaneous feedback, and would be more convenient. Since this parent (JS) had already developed a network of special needs families across Canada and the world, she partnered with another parent to see whether other parents were interested in supporting this idea. In less than 2 hours, more than 30 parents were interested in participating. While parents were keen to participate, it was also important to convince the researchers that this was a viable venture. Our parent made a presentation to the *CanChild* knowledge translation team, and this was taken to the entire *CanChild* team for approval. While not an overwhelming number of researchers were using Facebook, it was agreed to try it as a pilot project to be evaluated and revisited in 6 months.

### Evaluation Method

To evaluate this Web-based community, we collected and analyzed posts, likes, and comments in the group over a period from June 2014 to March 2015. In addition, we gathered data through a survey sent to all members (active or not) of the group.

#### Facebook Evaluation

We informally evaluated the Facebook group at 6 months, when the *CanChild* director agreed to provide further support and resources for the group with the mandate to provide a more formal evaluation. The formal evaluation took place from June 2014 to April 2015. To determine whether the Parents Participating in Research (PPR) group was successful from both the researchers’ and families’ perspectives, we evaluated the group using quantitative Facebook statistics of engagement and activity (eg, number of posts, likes, comments, and engaged members). We further analyzed the posts by family members and researchers to determine what broad topics or discussions areas were most frequently discussed.

#### The PPR Web-Based Survey

We used a voluntary, closed, online survey of PPR Facebook members for further evaluation. The institutional review board committee deemed a separate approval for the survey not to be necessary, as the survey was part of a quality improvement measure. In developing the survey, we used a participatory approach and asked for parent volunteers within the Facebook group to help formulate the questions. There were 5 iterations of the questionnaire. The participation of the other members in designing the survey was mediated through the group moderator, who forwarded the suggestions and requests anonymized to DR and OK. The final version consisted of 13 questions covering the aspects “member’s description,” “research literacy,” “safety of the group,” “motivation,” “perceived change,” and “future directions,” along with an open-ended section for respondents to provide comments. The survey was distributed using SurveyMonkey (Palo Alto, CA, USA), and the link was shared with the group members through multiple channels as posts, email, and direct messages. Multimedia Appendix 1 shows a copy of the survey. According to the Checklist for Reporting Results of Internet E-Surveys (CHERRIES) guidelines, the link provided allowed for only one response per Internet protocol address, no personal information was collected, and participation was voluntary [18]. The intention was to also reach those parents and researchers who joined the Facebook group but did not use it on a regular basis. Over the period of 1 month (March 2015) the moderator launched 3 reminder actions. No incentive was offered for completing the survey.

We analyzed quantitative data using frequency statistics. For the open-ended question “what would you change about the group?” 2 of the authors reviewed and coded responses into categories based on agreement. Quotes selected to include in the paper were chosen by consensus of all authors that were thought to represent an interesting perspective on the Facebook group that wasn’t captured in the quantitative portion of the survey.

## Results

### Building the Community

In starting this Web-based community, several decisions had to be made based on principles established for community-based research [19]. The first was that the community would be set up and run by the 2 parent moderators. They worked in consultation with *CanChild* and considered everything from choosing the type of group, to finding members, to setting rules of engagement and deciding on areas of discussion. Tutoring was also a factor to help many members of the research team understand how to use Facebook.

#### Private Versus Public

It was decided that a private (or secret) Facebook group be set up for the purpose of this advisory community, and it was named “Parents Participating in Research” (PPR). The rationale for making the group private was that it allowed moderators to control who was part of the group (members would have to be invited to join by an administrator, and posts would be seen only by other members within the group) and that the group would *not* be searchable (allowing for increased confidentiality of information shared by parents and researchers).

#### Rules of Engagement

To moderate the space and ensure a clear purpose, rules of engagement were developed (see Multimedia Appendix 2). All members were asked to read and agree to follow the guidelines set out before commenting in the forum. We provided a community document for this purpose, with the idea that we would revisit these rules on a regular basis to ensure that we were providing a safe and comfortable space.

#### Community Space

The PPR Facebook group launched in June 2014 with its first members (primarily those who expressed an interest in the initial Facebook post) invited into the group on June 10 and 11, 2014. After signing off on the rules of engagement, they were invited to introduce themselves (or their children) in either the community photo album or in the group timeline. This was done to foster a sense of community and to help us remember that there is indeed a person behind every question and response. While not mandatory, introductions were encouraged to promote participation and engagement.

#### Icebreakers

Icebreakers were topics introduced by the moderator and used to help stimulate conversation and establish rapport. Multimedia Appendix 3 shows an example of an icebreaker.

### Facebook Evaluation

As of April 1, 2015, the PPR Facebook page had a total of 96 members (2 parent moderators, 13 researchers/ *CanChild* members, and 81 family members). The majority of the members were female, but there were 11 male members (7 of whom were researchers). We estimated that 4 members left the group during the pilot stage of this project. Members were primarily located in Canada (with representation from Alberta, Saskatchewan, Manitoba, Ontario, and Quebec; 1 from the United Kingdom, and 1 from Australia).

#### Engagement

During the time period June 2014 to March 2015, a total of 432 posts were made (this figure only includes original posts, not comments generated from the posts). Breaking this figure down further, 155 (35.9%) of these posts were made by a moderator (averaging 77.5 posts per member), and 197 (45.6%) posts were made by parents (averaging 2.4 posts per member). Researchers accounted for 80 (18.5%) of the 432 posts, averaging 6.2 posts per member. There was an initial surge of members in the inception phase (approximately June 2014) when a large proportion of members (n=31, 32%) were added to the group. This influx of members was accompanied by a high level of engagement, with a total of 64 primary posts being made in the month of June (mean posts per month: n=42.9, range 20-64 posts). Another period of increased engagement occurred in November of 2014 (64 primary posts made), as that month featured a Family Engagement Day hosted at McMaster University by *CanChild*, celebrating its 25th anniversary. As indicated above, moderators restricted access to the group to ensure that the group remained manageable and the group was not searchable from the public Facebook domain.

Based on the number of views, as displayed by Facebook, posts were generally seen by all members of the group (indicating that members checked in frequently). Posts had a median of 3 likes (range 0-24) and 4 comments (range 0-113).

#### Families

While the purpose of the Facebook group was to connect researchers and parents of special needs children, the Web-based community also provided a private environment in which parents could discuss personal issues and interact with other families with similar experiences. Many discussions covering various topics were initiated, and during the 9-month analysis period, 197 (45.6%) were made by parents alone (excluding moderators and researchers). Among these posts, the topics that were most frequently talked about were childcare (eg, topics surrounding behavioral issues, difficulties communicating with professionals), education and school (eg, topics surrounding participation and inclusion at school), and diagnosis-specific posts (eg, obtaining an accurate diagnosis, seeking research or therapy for a specific diagnosis). Furthermore, parents who connected with the group reported many benefits, including feelings of belonging, that this was truly a community they could be proud to call their own. They reported pride in making a difference in research, even if indirectly, and repeatedly said that they felt that their ideas, thoughts, and experiences were validated, that sharing their stories was not futile. As a result of parents recognizing the need for clinicians and researchers to hear their stories, several parent members initiated the development of a book of stories, which they will compile and whose proceeds will go back into furthering research. Additionally, parents indicated that they were able to ask questions and access information and resources that they would not have otherwise found, from people they could trust to give them the right information.

#### Researchers

This Web-based community provided researchers with an opportunity to consult families of special needs children to get guidance and hear issues that are important to them. Examples of the type of requests were a call for parents to read and provide input on a parent resource being developed, to provide input on the logistics and content of a Family Engagement Day, and to express their interest in contributing as a partner in a grant proposal to a national funding agency. An additional benefit was that researchers were able to guide parents to credible resources that were relevant to their needs, a limitation that was outlined in previous Web-based communities [13,14]. Of the 80 posts made by researchers, 44 (55%) were posts linking parents to a variety of credible resources, including websites, news stories, videos, info graphics, and articles.

One example of the direct impact and meaningfulness of the group for both parents and researchers was a post from one mother who expressed her disappointment that many family members do not understand the needs and abilities of her child. Family members tend to give well-meant but hurtful advice that can lead to tension within the extended family. Other parents from our group suggested that writing up a short profile about her child may be helpful. The mother took that suggestion to heart, developed a beautiful profile of her child’s strengths, likes, and dislikes, and posted the profile for others in our group to review and comment on. Other members praised the idea and the approach of this mother, and it generated an important discussion, regardless of the underlying diagnoses of their children. It was noted that aspects such as attitudes, family supports, and the ability to participate are important aspects of the quality of life of children and their parents. This discussion overlapped with the interest of one of the researchers (OK) in using the International Classification of Functioning, Disability and Health (ICF), developed by the World Health Organization [20], to better describe needs of patients with chronic health conditions and disabilities. The profile created by the mother was a good example to illustrate how the needs of the child could be classified in terms of the ICF. After obtaining consent from the mother who posted the profile, we used an anonymous version of it in a grant proposal to illustrate the needs of families in sharing meaningful information about their child using the ICF [21].

### PPR Web-Based Survey Results

#### Members’ Description

With 49 of a possible 96 responders to the survey, the response rate was 51%. A total of 82% (n=40) of the responders indicated that they were participating in the Facebook group due to their personal experience with disability (parents) and 18% (n=9) due to their research experience (researchers). Approximately two-thirds of parents and researchers indicated that they read the posts on a daily basis.

#### Research Literacy

The parents were asked to rate their research knowledge on a scale from 1 (low) to 10 (high) for 2 time points: (1) a retrospective assessment of their knowledge when initially joining the group and (2) their current knowledge. The parents had a median value of 6 (responses ranging from 0 to 10) (n=40) at entry to the group, which had increased to 8 (with responses also ranging from 0 to 10) (n=40) at the time of filling out the survey.

#### Safety

To evaluate how safe the users felt participating in this group, we asked respondents to indicate to what extent they regulated what they posted. Among the parents, 23 (58%) indicated that they felt safe to post sensitive or personal information, 10 (25%) indicated that they regulated what they posted, and 4 (10%) indicated that they only read and did not post at all. A total of 3 parent respondents (7%) did not answer this question. Among the researchers, 5 of the 9 (56%) felt safe to post sensitive or personal information, 3 (33%) regulated what they posted, and only 1 (11%) read posts but did not post themselves.

#### Motivation

To understand our community’s motivation for participating in this group, we gave them 6 possible response options. Table 1 lists the responses from parents and Table 2 lists the responses from researchers, followed by quotes from the open-ended questions.

#### Parents’ Quotes

I never realized that as a parent I could make a difference. This group has given me the hope and proof that I can.

I have expanded my knowledge of childhood disability—which in turn has helped me make connections with other parents. Even if their disability diagnosis and experience is different than mine, I find it helpful to see things from their point of view. I think that may be key in learning how to advocate for change not just for my own child but for any child.

#### Researchers’ Quotes

I was not aware of the impact of the daily struggles that disabilities can have in the life of families. Many of the topics brought up in the group have not been brought up in the same way in clinical encounters.

I have also learned how eager and supportive families are of research and how willing they are to provide feedback on any issues.

#### Perceived Change

Members were asked if they had changed their behavior or attitude in any way as a result of participating in the group. Table 3 lists the parents’ responses and Table 4 lists the researchers’ responses.

**Table 1:** Parents’ motivation to join the Parents Participating in Research group (n=37).

Motivation	n	%
To connect with like-minded people	35	95
To find information, eg, search for or ask a question	29	78
To have an impact on childhood disability research	28	76
To get or give emotional support	27	73
To share ideas and solicit feedback	22	59
To raise awareness about issues related to disability	19	51

**Table 2:** Researchers’ motivation to join the Parents Participating in Research group (n=9).

Motivation	n	%
To have an impact on childhood disability research	9	100
To share ideas and solicit feedback	7	78
To connect with like-minded people	7	78
To raise awareness to issues related to childhood disability	6	67
To find information, eg, search for or ask a question	4	44
To get or give emotional support	4	44

**Table 3:** Parents’ perceived behavior and attitude changes after participating in the Parents Participating in Research advisory community (n=34).

Changes	n	%
No changes	19	56
Toward research	11	32
Toward their child/children	8	24
Toward their family	6	18
Toward people with disabilities	6	18
Toward their friends	5	15
Toward their patients	3	9
Other (eg, more aware of my child’s rights)	4	12

**Table 4:** Researchers’ perceived behavior and attitude changes after participating in the Parents Participating in Research advisory community (n=9).

Changes	n	%
Toward research	3	33
Toward their patients	2	22
Toward people with disabilities	2	22
Toward health care professionals	2	22
Toward their child/children	1	11
Toward their family	1	11
Toward their friends	1	11
No change	1	11
Other (eg, increased awareness of true engagement of parents in research)	2	22

#### What Would You Change About This Group?

Respondents were asked “if you could change one thing about this group, what would it be?” The answers were coded into themes by 2 authors (OK & DR). The 2 most frequently mentioned comments are summarized in the following 2 themes. First, more researcher input: 9 respondents mentioned that they would like to see more researchers actively involved in the group. They stated that they would like information on what research is being done, including what projects may require partnering, and they wanted researchers to engage stakeholders in discussion on *CanChild* material already posted on the *CanChild* website. In addition, they wanted researchers to respond quicker when tagged and join the discussions not only as a professional but also with multifaceted dimensions of themselves as a whole person. They wished that the researchers wouldn’t shy away from empathetic responses and talking from personal experiences, as well as presenting data and evidence-based information. Second, 5 comments were made suggesting the need to better clarify the purpose of the group, as there was a lot of discussion about patient care and family topics in addition to research topics.

## Discussion

Parent-led support groups have been found to serve a vital function in supporting families of children with disabilities [22,23] but are often specific to one health condition, rarely include other stakeholders such as clinicians or researchers, and are not framed to help move a research agenda forward. Likewise, research-initiated engagement activities are often limited in their scope; include limited number of individuals or voices, reflecting a potential biased view of the issue; focus only on a specific health condition; or only bring in families at strategic points in the research cycle (eg, at the end to disseminate the findings). Camden et al summarized strategies used in the past to recruit stakeholders in rehabilitation research (primarily people with disabilities and their families) as targeted (eg, by direct invitation to individuals) or open (eg, by asking partner organizations to solicit from their membership, or by using media) [10]. Most activities were done by committees and tended to be face-to-face meetings or teleconference meetings. Our approach, which was suggested by a parent, was to use Facebook as a useful, easily accessible way of actively engaging families in the research process.

*CanChild’s* overall mandate is to conduct clinically relevant research to improve the lives of children with disabilities and their families [24]. In order to fulfill this mandate, many of our research studies in the past have engaged youth with a disability or their family members as a collaborator and author (eg, The KIT “Keeping it Together,” Youth “KIT,” and Partnering for Change) [25-27]; however, we saw the opportunity to broaden our perspective by engaging a larger community of families to further address issues of importance to families, as well as create a community where there is an opportunity for ongoing meaningful dialogue.

The 4 stages of building an effective online health community as defined by Young are inception, establishment, maturity, and mitosis [16]. Using this framework, we describe the first year of our Web-based community, where we have moved through inception to having an established community

### Inception Phase

Key components of the inception phase are to invite members, build relationships, establish the tone and style of interaction, and nurture an active core membership [16]. The PPR Facebook group was proposed, launched, and moderated by a parent of a child with special needs who was acting as a parent resource to *CanChild*. She immediately recruited another parent to help cofacilitate the group and began strategies to connect members and begin to build trust. The initial purpose of the group was to be an advisory to *CanChild*, and 76% of parents and all the researchers responding to the survey identified that the reason for participating in this group was to have an impact on childhood disability research. Parents were also keen to connect with like-minded people and find information, while researchers wanted to connect with like-minded people, share ideas, and elicit feedback.

### Establishment Phase

When more than 50% of group content is generated by its members (as opposed to moderators), it is described as an established online community, while greater than 90% makes it a mature community [16]. The friendly icebreakers posted by the moderators were a safe and inviting way for people to begin sharing ideas, discuss common issues, and support each other. As time went on, the need for icebreakers was not as high, and members began to freely post discussion topics of their own. Members of the group (as opposed to moderators) generated 64% of initial posts, indicating that our group has transitioned into the established phase. With this shift it is important to recognize that the moderators still have an essential role to help ensure sustainability of the community [28]. The moderators readily respond to posts initiated by members or direct message, or tag others who may be able to add important perspectives to the discussion, ensuring that members feel heard and respected.

While a few researchers are active in this group, feedback from the survey highlighted the wish of parents for more researcher engagement, with ideas about what types of engagement would be welcomed.

An unexpected outcome was the shift in the emphasis of the group from acting primarily as an advisory to ongoing work at *CanChild*, to having a very active parent exchange where issues that are important to families readily came to the forefront. As one clinician researcher highlighted, the PPR Facebook group has provided a deeper understanding of what issues are important to families and the day-to-day issues they face, which don’t typically come up in clinic visits. This provides an opportunity to explore issues that may not have been thought of previously and to engage with participants to review the evidence and possibly develop the ideas into a research proposal. In a recently published study in the United Kingdom looking at research impact, Morton suggests it is not always possible to predict the impact that research partnerships will have at the outset, but that working closely with research users can help give a deep understanding of the users’ context, their actions to adapt research to their own needs, and the commitment to use research to make a difference [29].

Through the Facebook page, we had the opportunity to ask families for advice on a variety of issues (eg, topics and the format for *CanChild* ’s Family Engagement Day; improving our website to be more parent friendly) and to ask for feedback and collaborators on papers, evidence briefs, grants and presentations. We have 3 parents from our group as authors on this paper, and 1 on a recent review of stakeholder engagement [10]. Our moderator has copresented with our researchers at our provincial meeting of children’s rehabilitation organizations [30] and is providing a video to include in a panel discussion of family engagement at an upcoming international meeting. The moderator from our PPR group has participated in several *CanChild* research rounds, providing important family perspectives on a variety of issues.

### Maturity Phase: Strategies to Move Forward

It has been suggested that, in order to be successful, communities need to have a clear purpose, have a management strategy, and foster a sense of community [16]. Feedback from the survey indicated that, even though there are terms of reference for the group, the purpose of the group still needs further clarification. This may be because this group was initially set up as an advisory to *CanChild*, but the number of researchers participating in the group is low relative to the number of parents, allowing parents to continue to use the page in a manner that best meets their needs. To do community-based research, it is important that the researchers establish trust and demonstrate commitment, spending time in the community on an ongoing basis [13]. The few research members who are actively engaging with families feel a strong sense of open exchange and community. *CanChild* is actively trying to engage more researchers into the Facebook group; however, this remains a challenge, as researchers who are not regular Facebook users are reluctant to take the time to learn and worry about the ongoing time commitment it would require. Some researchers also struggle with their professional boundaries and knowing when and how they are to interact on a more personal level with families—even though this is what parents are asking for.

Since the survey, we have instituted a number of strategies to try to increase researcher engagement. There is now a Community and Family Engagement Officer at *CanChild* who will actively monitor the site and identify researchers with expertise who might be able to respond to parent posts, even if they aren’t active Facebook users. We have recently presented the results of the PPR Facebook evaluation at *CanChild* research rounds, providing examples of many of the interesting topics discussed, the impact the group has had on research members’ research (eg, the ICF example), and the request from parents for more researcher involvement. In addition, we have instituted a “meet the researcher” in our Facebook page to have a specific time that a researcher will be on the page to respond directly to parents’ questions. There is usually an introduction to the researchers’ area of research through a paper or news link prior to the meeting time. This has proved very successful in actively engaging members and introducing new researchers into the Facebook group. We also plan to act on ideas brought forward by families for more discussion on the content of our website.

We believe that several factors have contributed to the success of this group. The group’s growth from inception to an established community indicates the level of interest and engagement of its members. The importance of ongoing community conversations to maintain the interest and momentum of the group and engage members enough to feel safe to disclose personal information and provide advice cannot be underestimated. Since the moderators are parents of children with special needs who already had credibility with numerous parent groups was and still remains a real strength. Their knowing how to engage families and build a respectful, supportive environment while understanding the needs of the researchers and the overall purpose of the group were fundamental for the success of our group. The fact that the group welcomes families of children with a variety of diagnoses has allowed common issues to emerge, which are universal regardless of ability. The convenience that Facebook provides in terms of 24-hour accessibility was also seen as a positive for both busy parents and researchers as to when they can log in and participate.

### Limitations of the Study

The response rate to our survey was only 51%, which leaves us with just under half our members’ views not incorporated in the results. In addition, the survey was developed with the input of parent and researcher members but was not tested for reliability prior to its use. A validated tool to evaluate the Facebook community would have been very useful.

Another limitation was that our Facebook site was set up as a “group” in order to have the ability to be closed or “secret” and, in retrospect, this made harnessing accurate Facebook metrics a challenge. We tried purchasing Facebook reporting software but it was limited in its ability to provide accurate data from posts prior to purchasing it and we therefore needed to collect our data manually.

### Conclusion

The experience of being part of this Facebook group made participants aware of the need to invite youth with disabilities (in addition to parents) into the group or to organize a similar group to engage specifically with youth. The perspectives brought from the lived experience and the issues raised by youth would likely be quite different from the ones raised by their parents and are important for researchers to understand. This led to a focus group with 6 youth with special needs, and it became clear that they did not want to join the parent community but will move forward in developing their own community, which will provide opportunities to exchange ideas with *CanChild* researchers and each other. This is an example of what Young [16] might refer to as mitosis.

Young also suggested that the success of Web-based communities depends on having sustained organizational support in terms of financial and human resources [16]. Based on an initial positive review of the Facebook group at 6 months, *CanChild* has successfully applied for project funding to ensure sustainability of the group and allow financial support for the parent moderator with the goal to build a Web-based community in partnership with a national center of excellence for neurodevelopmental disabilities in Canada (NeuroDevNet, 2015-2018). We will use the results of this evaluation to help improve the Facebook page to meet the needs of *CanChild,* NeuroDevNet, and the PPR members as we work together to identify needs, important research questions, and actions to improve the lives of children and their families.

By acknowledging the benefits and being cognizant of the limitations of social media platforms, researchers can begin tapping into the potential for social media to be used as a means of engaging parents and families in the research process. Families can connect with other families and researchers to share their experience and voice what is important to them, to ensure that research is meaningful and impactful for those who needed it most: the children and the families.

## Appendix

Multimedia Appendix 1 Parents Participating in Research online survey.

Multimedia Appendix 2 Rules of Engagement for the Parents Participating in Research Facebook Community.

Multimedia Appendix 3 Screenshot of a sample icebreaker from the Parents Participating in Research Facebook Community.

## Abbreviations

CHERRIES: Checklist for Reporting Results of Internet E-Surveys
ICF: International Classification of Functioning, Disability and Health
PPR: Parents Participating in Research
