# Supplementary material for: Knowledge Exchange and Discovery in the Age of Social Media: The Journey From Inception to Establishment of a Parent-Led Web-Based Research Advisory Community for Childhood Disability
Source: J Med Internet Res. 2016 Nov 11;18(11):e293. doi: 10.2196/jmir.5994 (PMC5124112; doi:10.2196/jmir.5994)
Supplement: Multimedia Appendix 1 [file jmir_v18i11e293_app1.pdf]

Parents Participating in Research Facebook Group

Engagement

You are receiving this survey as a member of the Facebook Group "Parents Participating in Research". We would like to know how this group is doing and how we could move forward. It should take you about 10 minutes. The results will be shared with all participants and serve as a baseline for future surveys. Thank you for taking the time to share your ideas!

You might participate in this group in different roles, depending on the topic that is being discussed (parent of a child with disability, person with a disability, researcher, friend of people with disability etc.). When you first heard about this group, what was the main reason that made you decide to join?

- ○ Due to my personal experience with childhood onset disability
- ○ Due to my research interests in childhood onset disability

Parents Participating in Research Facebook Group

Research

How confident/knowledgeable did you feel in understanding research before joining this group?

not at all									very confident/knowledgeable
◯	◯	◯	◯	◯	◯	◯	◯	◯	◯

Parents Participating in Research Facebook Group

How confident/knowledgeable do you feel in understanding research now?

not at all									very confident/knowledgeable
○	○	○	○	○	○	○	○	○	○

Parents Participating in Research Facebook Group

What best describes your use of this Facebook group?

	never	once a month or less	every other week	weekly	almost daily
I read the posts/comments	○	○	○	○	○
I like posts/comments	○	○	○	○	○
I comment	○	○	○	○	○
I post	○	○	○	○	○

If you responded "once a month" or "never" please tell us what might increase the frequency of your actions?

Parents Participating in Research Facebook Group

Experience

Please tell us what your expectations were when you joined this group?

Parents Participating in Research Facebook Group

Do you feel this group is a safe place to discuss health related or research related topics?

- ◯ Yes, I feel safe to post sensitive/personal information
- ◯ I regulate what I post
- ◯ I do not post, only read

Please explain

Parents Participating in Research Facebook Group

Do you feel supported by the other members in this group?

lack of support — fully supported

( ) ( ) ( ) ( ) ( )

How would you describe the activities of the moderators of the group?

	not at all	too little	just the right amount	too much
Engaging members	◯	◯	◯	◯
Posting new topics	◯	◯	◯	◯
Refocusing discussions	◯	◯	◯	◯
Summarizing discussions	◯	◯	◯	◯

Any comments or suggestions? Please let us know!

Parents Participating in Research Facebook Group

Tell us a bit about why you are using this Facebook group! Please check all that apply and feel free to comment!

- [ ] to find information, e.g. search or ask a question
- [ ] to share ideas and solicit feedback
- [ ] to raise awareness about issues related to disability
- [ ] to connect with like minded people
- [ ] to get/give emotional support
- [ ] to have an impact on childhood disability research

Comments

Parents Participating in Research Facebook Group

Would you recommend this group to other parents, friends or colleagues?

- ○ Yes
- ○ No

Comments

Parents Participating in Research Facebook Group

As a result of joining this group what have you learned?

- [ ] ideas/strategies for advocating for my child at school
- [ ] ideas/strategies for advocating for my child with family and friends
- [ ] ideas/strategies for advocating for my child with healthcare professionals
- [ ] ideas/strategies for myself as a parent of a child with disability
- [ ] ideas/strategies for myself as a person with disabilities
- [ ] better understanding of research or the research process
- [ ] new ideas for future research
- [ ] broadened my perspective on the experiences of families

Comments

Parents Participating in Research Facebook Group

As a result of joining this group, have you changed your behaviour or attitude in any way in relation to:

- [ ] your child/children?
- [ ] your family?
- [ ] your friends?
- [ ] your patients?
- [ ] people with disabilities?
- [ ] healthcare professionals?
- [ ] research?
- [ ] other
- [ ] no changes

Please tell us one thing that you have changed as a result of participating in this group?

Parents Participating in Research Facebook Group

The Future

If you could change one thing about this group, what would it be?
