# Supplementary material for: Knowledge Exchange and Discovery in the Age of Social Media: The Journey From Inception to Establishment of a Parent-Led Web-Based Research Advisory Community for Childhood Disability
Source: J Med Internet Res. 2016 Nov 11;18(11):e293. doi: 10.2196/jmir.5994 (PMC5124112; doi:10.2196/jmir.5994)
Supplement: Multimedia Appendix 2 [file jmir_v18i11e293_app2.pdf]

Multimedia Appendix I- Rules of Engagement for the Parents Participating in Research Facebook Community

Rules of Engagement for Parents Participating in Research- ***both parents and the research team must agree to the following terms before participating in this group.***

1. **Treat people the same as you would face-to-face.** In other words it is easy to hide behind the computer. In some cases it empowers people to treat others in ways they would not in person. Remember there is a person behind the name on your screen. Treat all with dignity and respect and you can expect that in return.

2. **Please remember not to share sensitive information about yourself or your family while engaging in this space**. Moderators are not responsible for the outcome of any such action and we encourage you to exercise your discretion. If you are not comfortable sharing something, don’t!

3. **While we encourage open information sharing between parents/caregivers/advocates and the research team, please note that due to legal concerns there will be NO MEDICAL advice** given. Please do not ask or expect it.

4. Currently, due to privacy concerns with both the parent/caregiver/advocates information AND the research we may be discussing our group is set to "secret" which means that you will not be able to "share" the posts you see here with your Facebook friends. Members must be added by the group moderators. **Nothing that you post (photos, personal information that share, comments) may be used in any public manner without your expressed consent. If you are asked for permission you ARE NOT OBLIGATED to agree to it. It is entirely up to you what you decide to share!**

5. **We reserve the right to regularly and extensively review our Rules of Engagement, and update them if we feel that changes are warranted.** We will do our best to make sure that all members are kept up to date with any changes that are made.

6. **As a member of 'Parents Participating in Research' I understand that researchers, including student research assistants, may be added at any time to collect data pertaining to discussion topics that might inform the usefulness and development of the group.** *Data could be used in publications, but* without identifying any individuals. We feel that it is important to publish what the group sees as important ideas and may need some quotes as well to support points. By agreeing to the Rules of Engagement, you are giving permission to do this. (added October 18, 2015)

Please comment below to show that you have read and agree to follow the Rules of Engagement before commenting on any posts.
