# Supplementary material for: Knowledge Exchange and Discovery in the Age of Social Media: The Journey From Inception to Establishment of a Parent-Led Web-Based Research Advisory Community for Childhood Disability
Source: J Med Internet Res. 2016 Nov 11;18(11):e293. doi: 10.2196/jmir.5994 (PMC5124112; doi:10.2196/jmir.5994)
Supplement: Multimedia Appendix 3 [file jmir_v18i11e293_app3.pdf]

Multimedia Appendix II- Screenshot of a sample icebreaker from the Parents Participating in Research Facebook Community

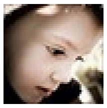

Jenn Sprung

14 June at 10:58

*****Weekend Ice Breaker!!*****

Leisure activities are a right of passage for most kids- and they can be anything from participating in sports, enjoying cultural activities (like museums, festivals and concerts) music or art lessons or even a trip to the local park. Do you think that children with special needs have equal access to leisure activities? Does your community support inclusive activities for children? Do your children participate in any organized activities? If they do, which ones? If they don't, what are the barriers that you have encountered? (e.g For us, it tends to be financial aspect, and that while Owen CAN participate, he is far behind his peers, and would require extra support that isn't available. For other families, it may be that adapted equipment is far beyond reasonable in cost)

Like · Comment

[redacted]ikes this. ✓ Seen by 35

View previous comments 3 of 53

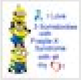

[redacted]..The city also offers music in the park - mostly local talent....but for the music lovers, there's room to dance and move around or to set up a blanket or lawn chair if you want....I think Belleville used to offer this at one point, not sure if they still do???

20 June at 10:04 · Unlike · 1

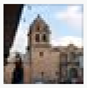

[redacted]A parent sent me this link to a resource on swimming which she found very useful. Thought I would share:
http://www.swimming.org/.../Inclusion_of_swimmers_with_a...

20 June at 14:55 · Edited · Like
